# First isolation and molecular characterization of *Toxoplasma gondii* strains from human congenital toxoplasmosis cases in Monastir, Tunisia

**DOI:** 10.1038/s41598-020-59060-w

**Published:** 2020-02-06

**Authors:** Ibtissem Lahmar, Arwa Lachkhem, Oussama Babba, Darine Slama, Aida Trabelsi, Karine Passebosc-Faure, Marie Laure Dardé, Hamouda Babba

**Affiliations:** 1 Laboratoire de Parasitologie–Mycologie Médicale et Moléculaire (code LR12ES08), Département de Biologie Clinique B, Faculté de Pharmacie de Monastir, Université de Monastir, Monastir, Tunisia; 2 Centre de Maternité et de Néonatologie de Monastir, Monastir, Tunisia; 3 Centre National de Référence (CNR) Toxoplasmose/Toxoplasma Biological Center (BRC), Centre Hospitalier-Universitaire Dupuytren, Limoges, France; 4 Université de Limoges, Faculté de Médecine, INSERM UMR 1094, Neuroépidémiologie tropicale, Limoges, France

**Keywords:** Parasite biology, Parasite biology, Epidemiology, Epidemiology

## Abstract

*Toxoplasma gondii* is a protozoon parasite that can cause severe clinical problems such as congenital toxoplasmosis. The distribution of *T. gondii* genotypes varies from one geographic area to another. So far, little is known about the parasite genotypes in Tunisia, North Africa. The present study aimed isolating and genotyping *T. gondii* from the amniotic fluid (AF) and placenta of pregnant women in Monastir, Tunisia. Amniotic fluid and/or placenta from 80 women who acquired toxoplasma infection during pregnancy were tested by PCR and/or mouse bioassay. Genotyping of *T. gondii* isolates from these samples was performed with 15 microsatellite markers. Four viable T. gondii strains were isolated from either the AF or placenta of four women. Specifically, strains TUN001-MON1 and TUN002-MON2 were isolated from both the AF and placenta, TUN003-AHA from only the placenta, and TUN004-NEL from only the AF. The four viable strains were not virulent for mice. Genotyping revealed that the four strains were type II strains. This is the first report on isolation and genotyping of *T. gondii* from AF human samples in Tunisia. Further studies focused on *T. gondii* genotyping on a larger number of human cases and on animals in Tunisia are needed to improve the knowledge and epidemiology of toxoplasmosis.

## Introduction

*Toxoplasma* infection is one of the most prevalent parasitic disease, caused by the intracellular protozoa, *Toxoplasma gondii*. This protozoan affects all warm-blooded animals, including humans^1^. Humans are infected by the ingestion of Toxoplasma oocysts in water, food or cat feces polluted soil, or toxoplasma cysts present in raw or undercooked meat^2^. Congenital toxoplasmosis (CT) may happen following maternal primary infection during pregnancy. Risk of transmission increases with the pregnancy age, while severity of the disease for the fetus decreases. In fact, placenta barrier is more efficient at the first semester of gestation, allowing the passage of parasites in less than 10% of infected pregnant women. However, it becomes more permeable during pregnancy evolution, leading to parasite transmission around 30% and 60–70% of infected pregnant women in the second and third trimester respectively^3^. Although, most congenitally infected newborns appear to be healthy at birth, they may develop symptoms until months, years, or even decades later in life. Hydrocephalus, intracranial calcifications, mental retardation, hepatosplenomegaly, and chorioretinitis are classical signs associated with the disease^3^. Undiagnosed and untreated patients may expand irreversible lesions, particularly brain calcifications, hydrocephalus, and eye disease^4^. Severity of CT may be associated to several factors including parasite genotype, host genetic variability and immune response^5–7^. Previously, *T. gondii* complex was classified into three major lineages designated type I, II and III^8^. However, recent studies using various molecular tools and analyzing genetic polymorphism of T. gondii have revealed a larger genetic diversity including atypical and recombinant genotypes^9–11^. Majority of studies conducted in Europe reported that more than 80% of Toxoplasma strains isolated from infected human belongs to genotype II^12,13^. However, genotypes I and III are responsible for only some sporadic cases^14^. So far, little is known about genetic diversity of *T. gondii* strains in Africa^15,16^. Nonarchetypal genotypes (e.g. Africa 1 and 2) were isolated in West and Central Africa^14,17^, while type II and III were reported in North and East Africa^18^. Even though, *T. gondii* was firstly discovered in Tunisia in 1908 from *Ctenodactylus gundi* tissues^19^, data concerning parasite genotypes distribution in animals and human are still missing. To the best of our knowledge, only a single study reported the isolation of *T. gondii* from the placenta of a fatal CT case in Tunis (North of Tunisia)^20^. However, no study succeeded to isolate parasite from AF. In addition, only few studies were conducted on genotypic characterization of *T. gondii* in Tunisia^20,21^. By using PCR-RFLP multilocus analysis, these studies showed that most Tunisian cases were a mixture of type I/II or I/III alleles.

Thus, the aims of this study were to isolate *T. gondii* from the AF and the placenta of pregnant women in Tunisia (Monastir governorate), and characterize the isolates using 15 microsatellite markers.

## Results

### PCR Analysis and strain isolation

Toxoplasma PCR was positive in only three over 67 AF (TUN001-MON1, TUN002-MON2, and TUN004-NEL) and in two over 43 placentas (TUN002-MON1 and TUN002-MON2). The microscopic examination of brain tissue from seropositive mice six weeks post-inoculation revealed the presence of *T. gondii* cysts in brain mice inoculated by amniotic fluid and/or placenta (TUN002-MON1, TUN002-MON2, TUN003-AHA, and TUN004-NEL). TUN003-AHA strain, isolated from a woman infected in the third gestational trimester, was positive only in mouse bioassay (Table 1). All the isolated strains were non virulent for mice.

**Table 1:** Clinical and biological data for *T. gondii* infected women and their newborns.

Isolate	Prenatal diagnosis				Postnatal diagnosis				Post natal follow-up	
	Date of SC (WA)	Ultrasound	Amniotic fluid analysis		Placenta examination		Western blotting^b^		Serology^c^ at 1 yr of life IgG	Clinics
			PCR	mouse inoculation	PCR	mouse inoculation	IgM	IgG		
TUN001-MON1	25	N	pos	pos	pos	pos	pos WB	pos WB	+	N
TUN002-MON2	26	N	pos	pos	pos	pos	pos WB	pos WB	+	Chorioretinitis
TUN003-AHA	29	N	ND	ND	neg	pos	neg WB	id WB	−	N
TUN004-NEL	25	N	pos	pos	neg	neg	neg WB	id WB	−	N

^a^Abbreviations: SC, seroconversion; WA, weeks of amenorrhea; ND, not done; N, asymptomatic; pos, positive; neg, negative; yr, years, ^b^, neg WB, absence of synthesis IgM antibodies; pos WB, neosynthesis of IgG antibodies; id WB, identical profiles ^C^, (+) increased IgG antibodies after birth; (−) decreased IgG antibodies after birth.

### Postnatal follow-up

**Figure 1:**
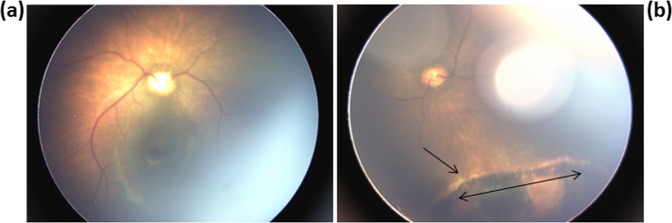
Fundus photography, (**a**) Normal fundus, (**b**) Peripheral chorioretinal lesion in the left eye (arrows).

The diagnosis of CT was defined if at least one of the following tests was positive (i) *T. gondii* PCR and/or mouse inoculation with amniotic fluid prenatally or at delivery, or in placenta, (ii) detection of specific Toxoplasma IgM antibodies after three days of birth, (ii) persistence of specific IgG until 1 year of age, (iii) different immunoblot profiles of anti-*Toxoplasma* IgG and/or IgM antibodies between the mother and the newborn at birth. In our findings, neonatal diagnosis of CT by western blotting showed identical profiles of IgG antibodies in the serums of the mother and newborns and absence of IgM antibodies in newborns. For only one child who was infected with TUN002-MON2 showed a neosynthesis of IgG and IgM antibodies and an increase of the levels of IgG antibodies after birth. Furthermore, ophthalmological examination was performed for the four born-infants in the first months after birth. Among them, only one child had a peripheral chorioretinits scar in the 1^st^ month old (Fig. 1), while the three others were asymptomatic and the eye fundus were all normal within the first six months of life (Table 1).

### Microsatellite genotyping

When compared to reference strains, the four *T. gondii* strains were identified as type II strains (Table 2). The *T. gondii* strains were banked at the *Toxoplasma* Biological Ressource Center under the Accession Number TgH 104001 A, TgH 104002 A, TgH 104003 A and TgH 104004 A.

**Table 2:** Genotyping results of *T. gondii* DNA with 15 microsatellite markers for the 4 isolates collected in amniotic fluid and/or and from 4 reference strains.

Isolate (Genotype)	Origin	Microsatellite marker (size; base pair)*														
		*B18*	*M33*	*TUB2*	*XI.1*	*TgM-A*	*W35*	*IV.1*	*B17*	*M48*	*M102*	*N60*	*N82*	*AA*	*N61*	*N83*
GT1 (Type I reference)	Goat	160	169	291	358	209	248	274	342	209	168	145	119	265	87	306
PRU (Type II reference)	Human	158	169	289	356	207	242	274	336	209	176	142	117	265	123	310
VEG (Type III reference)	Human	160	165	289	356	205	242	278	336	213	188	153	111	267	89	312
DPHT (Africa 1 reference)	Human	160	165	291	354	205	248	274	342	225	166	147	111	271	89	306
TgH 104001 (Type II)	AF	158	169	289	356	207	242	274	336	215	182	140	109	265	99	310
TgH 104002 (Type II)	AF/P** PPPP	158	169	289	356	207	242	274	336	215	184	142	109	275	103	312
TgH 104003 (Type II)	P	158	169	289	356	207	242	274	336	215	184	140	109	275	103	312
TgH 104004 (Type II)	AF	158	169	289	356	207	242	274	336	215	184	140	109	295	113	312

*The first 8 markers are used for type characterization,

**P: placenta.

## Discussion

The isolation of the parasite by inoculation in the mouse has a great contribution in the confirmation of the diagnosis of congenital toxoplasmosis. Also, this method makes possible to isolate live strains of the parasite which can be used later in epidemiological and clinical studies. In addition, *T. gondii* can be isolated from different biological samples (AF, placenta, cord blood, cerebro spinal fluid). In Tunisia, only one study isolated the live parasite from the placenta^20^. In our study, we succeed to isolate the parasite from the placenta and/or AF from four seroconverted women during pregnancy. According to our knowledge, this is the first isolation of live *T. gondii* strains from AF samples in Tunisia. Furthermore, we identified the genotype II in all isolated strains which were not virulent for mice in inoculation assays.

Contrarily, previous studies published in Tunisia, identified other genotypes. Indeed, it is reported by Boughathass *et al*., (2010) that direct genotypic characterization of *T. gondii* strains from congenital toxoplasmosis cases by using PCR-RFLP multilocus analysis showed that most Tunisian strains were a mixture of type I/II or I/III alleles^20,21^. Moreover, the same team revealed in the next year (2011) an atypical genotype of *T. gondii* with unusual genetic composition in amniotic fluid from a diabetic pregnant Tunisian woman^22^.

Regarding the *T. gondii* genotyping on animal’s cases performed in Tunisia, genotype analysis reveals the considerable proportion of type III (55%), followed by recombinant genotypes (22%) and only 11% for type I and type II strains on slaughtered sheep^23^. Such results suggested that the sheep might be the source of human contamination by ingestion of cysts in undercooked meat.

Our results of genetic characterization of *T. gondii* type II are in agreement with those observed in Algeria, bordering Tunisia to the North-East, in where the same type was isolated from two human congenital toxoplasmosis cases^24^. Similarly, in the same country, it has been revealed that 12 of 22 isolates in stray cats mainly belonged to the type II lineage^25^.

Nevertheless, it may be thought that neighboring countries with old trade relations can host the same type of strains. Thus, the intense trade in various foodstuffs, a potential source of toxoplasma, between Algeria and Tunisia, may explain why the same genotype II is found on both shores of the Mediterranean. In Africa, and particularly, in Egypt, Abdel Hameed and Hassane in (2008) using a n-PCR-RFLP of a single polymorphic marker found *T. gondii SAG2* type II in 87% of 38 cases with abortion and intra uterine fetal death suggesting the possible role of type II in these cases^26^. Type II also dominates the genetic diversity in domestic animals in Ethiopia^27^.

Elsewhere, in European countries it has been shown that more than 80% of strains isolated from human infections belong to genotype II^12,13,28,29^. Genetic characterization of samples from German patients with cerebral, ocular, and systemic toxoplasmosis was achieved in 88% of *T. gondii* type II. Similarly, in Germany, the predominance of *T. gondii* type II oocysts isolated was found in cats and in tissues of other intermediate hosts^30^.

The severity of the congenital disease is influenced by the time of gestation at which the mother became infected as reported in several studies^3,31^. Furthermore, genotype of *T. gondii* could contribute to the pathogenicity of the disease. Our results are in agreement with these notions since, the three of four neonates are mothers infected at the end of second trimester, are born asymptomatic. While, only one has unilateral peripheral retinal lesion. This could be due to early treatments that reduce the severity of the disease. Nevertheless, the isolation of *T. gondii* from placenta, without detection of a congenital infection, is quite exceptional: in our study only one such cases (TUN003-AHA), was reported in the management of four *T. gondii* infections occurring during pregnancy. This is in agreement with other studies who reported that placental infection may not systematically involve a fetal infection^32,33^. Moreover, in our study, the rate of CT in Monastir is about 0.03% (4 cases/10,560 births). Our results are in agreement with the study carried out in South of Tunisia by Sellami *et al*.^34^. In fact, a positive rate of 0.04% has been found (16/40,566). However, in North of Tunisia Boudaoura *et al*.^35^ reported 35 CT cases among 6,074 suspected toxoplasmosis infections.

At concluding remarks, this study presents the first report of *T. gondii* strains isolation in Tunisia from human AF samples. Also, all isolates were belonging to type II genotype. More studies focused on *T. gondii* genotyping on a large scale of human and animal populations are required to improve the knowledge of toxoplasmosis epidemiology.

## Materials and Methods

### Ethics statements

The study was carried out according to the Declaration of Helsinki Principles and all Tunisian pertinent regulations. The samples were obtained for routine diagnostic purposes from pregnant women who were managed at the Centre of Maternity and Neonatology of Monastir at the request of the gynecologist. We confirmed that informed consent was obtained for all subjects. In fact and during consulting, the gynecologist informs pregnant women of the importance of pre and post-natal monitoring of toxoplasmosis that required biological analysis of amniotic fluid, placenta and blood. Given the seriousness of the situation the pregnant woman is convinced of the importance of this diagnosis for her health as well as that of her baby. After acceptance, the gynecologist prescribes a request for analysis containing the different information (age of the patient, age of pregnancy, date of seroconversion, origin,).

### Seroconversion diagnosis

**Figure 2:**
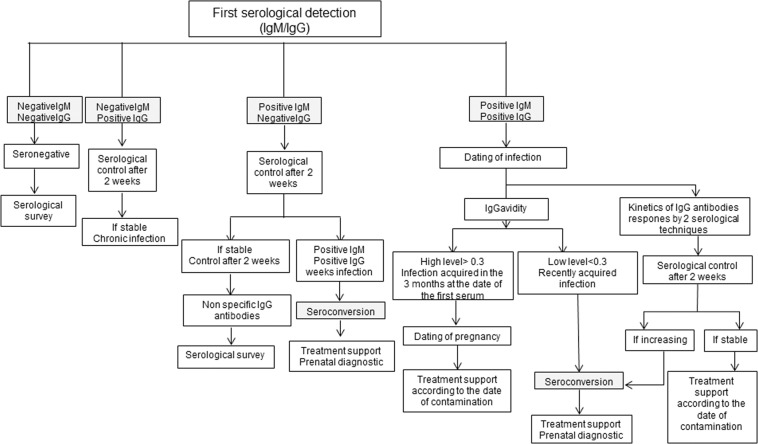
Chart of screening pregnant women with toxoplasmosis in Monastir.
  Seroconversion was confirmed by the appearance of IgG with positive IgM after an initial negative IgG and IgM sample or higher titer in IgM than the previous one with negative or slightly positive titer of IgG antibodies and low avidity index.
  All these cases were treated with spiramycin at the standard dosage of 9 × 106 units per day until delivery and underwent monthly echographic follow up.
(ii) Prenatal diagnosis. Amniocentesis was performed after 16 weeks of gestation and at least 4 weeks after maternal infection^36^.
  Prenatal diagnosis was attempted for 67 AF collected from 80 women. The remaining 13 women either did not consent (n = 10) or seroconverted late in the third trimester (n = 3). These AF were tested for *T. gondii* by PCR and mouse inoculation. If the results by one of these techniques were positive, women received treatment with pyrimethamine plus sulfadiazine with folinic acid supplementation as permitted in Tunisia.
(iii) Postnatal diagnosis. At delivery the placenta (n = 36) and/or the neonatal AF (n = 7), at the time of amniotic sac rupture, were obtained and tested for *T. gondii* detection by PCR and mouse inoculation. Neonatal and maternal serological profiles were compared using a commercial test western-blot IgG/IgM (LDBio Toxoplasma WB IgG/IgM Kit (LDBio Diagnostics, Lyon, France) following the manufacturer’s recommendations. The band pattern obtained for IgG and IgM within coupled newborn and mother’s serums was analyzed and compared in terms of number of bands and intensity. The presence, on the newborn’s strip, of bands that were not detected in the mother’s serum or that were more intense was considered indicative of neosyntetized antibodies production. Serological profile of the newborn was checked after one month from delivery and then every three months until disappearance of maternal IgG.

(i) Patients. This retrospective study was carried from 2012 to 2017. It included 10,565 pregnant women who were routinely diagnosed and managed at the Centre of Maternity and Neonatology of Monastir, Tunisia. From these, 80 women acquired toxoplasmosis infection during pregnancy. These later were originated from the neighboring cities of the governate of Monastir. The mean age of the seroconverting women was 29 years (rang: 19 to 46).
  Serological monitoring was assessed for the detection of anti-*T. gondii* IgM/IgG antibodies with the following assays: the Vidas Toxo system (BioMérieux), the Platelia Toxo IgG/IgM (Bio-Rad), and the Immulite (Siemens). The Vidas Toxo IgG Avidity assay (bioMérieux) was used for the estimated time of infection (Fig. 2).

Newborns were treated with spiramycin until congenital toxoplasmosis was ruled out. Infected babies were treated with pyrimethamine and sulfadiazine with folinic acid supplementation.

#### PCR diagnosis

Antenatal or neonatal AF samples (20 ml) were centrifuged at 1,500 × g for 10 min. The pellet was suspended in 7 ml of AF and kept for the injection to mice. Placenta was cut into approximately 2 × 2 cm cubes, weighed (5–100 g) and blended in a food processor. The crushed tissue was digested using a 0.25% trypsin (Gibco) in a sterile saline solution. The digestion was performed at 37 °C for 1.5 h with continuous stirring. Subsequently, digested tissue was filtered through sterile gauze and washed three times using a sterile normal saline solution. After a final centrifugation for 10 min at 1.500 × g, obtained pellet was resuspended in 10 ml of normal saline solution supplemented with penicillin (1000 U/ml). DNA was extracted in duplicate from the pellet with the QIAamp DNA miniKit (Qiagen). A PCR targeting a 529-pb repetitive DNA element of *T. gondii* was performed in duplicate. Reaction mixture (50 µl) contains 10 µL of DNA template, 2 µM of each primer, 200 µM of dNTP (invitogen) and 0.25 U Hot Start Taq DNA Polymerase (Qiagen). Amplification was performed in Perkin Elmer Thermo cycler (GeneAmpPCRsystem^TM^ 2400) using the following conditions: an initial denaturation for 15 min at 94 °C followed by 40 cycles of 30 s at 95 °C, 30 s at 58.8 °C, 30 s at 72 °C and a final extension for 5 min at 72 °C^37^. PCR products were analyzed by electrophoresis in 2% agarose gel stained ethidium bromide.

#### Bioassay in mice

In order to isolate *T. gondii* strains, five 6–8 weeks-aged outbred Swiss-Webster mice, were intraperitoneally inoculated with 1 ml of patient sample (centrifuged AF, neonatal AF or placenta tissue digest pellet, supplemented with penicillin). After 6 weeks, blood samples were collected from mice by retro-orbital puncture. Mice were then sacrificed and their brains were harvested and homogenized with 1 ml of saline solution. Mixture was used for the microscopic detection of *Toxoplasma* cyst and for DNA extraction and PCR.

### Serology in mice

Indirect fluorescent antibody test (IFAT) was performed to detect specific antibodies to T. gondii in mice. Briefly, RH strain tachyzoites were used as antigen. Mice sera were diluted in phosphate buffer solution (PBS, pH 7.2) starting at 1:20 to 1:160 (cut-off at 1:20 dilution titer) dilutions and incubated at 37 °C for 30 min. Then the slides were probed with fluorescein isothiocyanate conjugated goat anti-mouse IgG diluted 1/100 in a PBS solution with 1% Evans blue (bio-Mérieux SA, France). Finally, the slides were washed with PBS and examined with a fluorescence microscope (LEICA DFC 310 FX). Positive and negative sera were used as controls.

### Multiplex-PCR microsatellite genotyping

DNA was extracted from mice brain using the QIAamp Tissue kit (Qiagen) according to the manufacturer’ instruction eluted in 50 µL of the elution buffer and stored at −20 °C until used for genotyping. *Toxoplasma gondii*. Strains were genotyped using 15 microsatellite markers distributed on 11 of 14 chromosomes in a single multiplex PCR-assay, as described previously^28^. The sizes of the alleles in base pairs were estimated using Gene Mapper analysis software (version 4, Applied Biosystem). Microsatellite profile of 4 reference strains belonging to type I (GT1), type II (PRU), type III (VEG), and Africa 1 (DPHT) are compared with those of strains isolated in this study. This work was made in collaboration with the Centre National de Référence (CNR) Toxoplasmose/Toxoplasma Biological Center (BRC), Centre Hospitalier-Universitaire Dupuytren, Limoges, France.

### Ethical issues

This research study has been approved by the Ethics committee of the Monastir Medical Faculty (IORG 0009738 N°21/OMB 0990-0279). All procedures were carried out in accordance with the European Union Regulations (Directive 2010/63/EU) for animal experiments and approved by the local Experimental Animals Ethics Committee of the Faculty of Pharmacy of Monastir
